# Welfare policy and suicide: The role of “supporting the self-reliance of persons in need” program in Japan

**DOI:** 10.1016/j.ssmph.2025.101852

**Published:** 2025-08-19

**Authors:** Xuanzi Zuo

**Affiliations:** Osaka School of International Public Policy, The University of Osaka, 1-31, Machikaneyama-cho, Toyonaka, Osaka, 560-0043, Japan

**Keywords:** Suicide, Welfare policy, Personal consultation, Cash benefit, Active labor market policy, Difference-in-differences

## Abstract

**Background:**

Japan experienced a substantial decrease in suicide rates in the 2010s. During this period, the “Supporting the Self-Reliance of Persons in Need” program was initiated to target individuals struggling to meet basic needs. The program provides personalized consultations and subprograms that address the residence issues, necessities, employment needs, and household finance management problems. This study evaluates whether this program is related to the decline in Japan's suicide rates in the 2010s.

**Methods:**

The first analysis employs a difference-in-differences approach, using the monthly suicide rates in 815 cities across Japan from 2009 to 2015. Policy exposure refers to the period after pilot programs were introduced in cities between 2013 and 2015. The second analysis examines whether the number of subprograms application counts is associated with the suicide rates by fiscal year.

**Results:**

The introduction of self-reliance support in cities is related to a monthly reduction of 0.066 and 0.041 in suicide rates among men and women, respectively. The associations are found among the population under 60 years, particularly strong for males and the middle-aged. The second analysis suggests that the employment training program shows the strongest association with lower suicide rates. An additional employment training application per 100,000 population is associated with a 0.256 reduction for males and 0.169 for females in the annual suicide rates.

**Conclusions:**

The introduction of self-reliance support is associated with reduced suicide rates, mainly among males and the middle-aged. The employment training subprogram demonstrates the strongest association with lower suicide rates.

## Highlights

• “Supporting the Self-Reliance of Persons in Need” is linked to reduced suicide rates.
• The employment training subprogram is associated with suicide reduction.
• Middle-aged males experienced the most substantial decline in suicide rates.
• Difference-in-differences analysis is used to analyze.
• Personalized economic support may complement traditional suicide prevention efforts.

## 1 Introduction

Existing research shows that welfare generosity can mitigate the impact of economic hardships such as unemployment, indebtedness, and financial difficulties on suicide. Theoretically, Durkheim proposed the social integration theory to explain the potential role of welfare policies in reducing suicide. Welfare policies represent the norms of mutual aid and support, which prevent isolation and thus reduce suicide (Durkheim, 1951; Kim, 2018; Zimmerman, 2002). Another well-acknowledged theory is the materialist approach, including 3 paths to prevent suicide. First, the welfare policy provides materials to maintain the well-being of the vulnerable. Second, the financial support will protect marriage and relationships around the vulnerable, which are the protective factors of suicide. Furthermore, the welfare policy can reassure the mental health of those with a high risk of economic problems before the situation worsens (Flavin & Radcliff, 2009; Kim, 2018). As empirical studies, the total welfare expenditure (Shiroyama et al., 2021; Tuttle, 2018) and spending on specific welfare programs—such as the active labor market (Mattei et al., 2019; Reeves et al., 2015; Stuckler et al., 2009) and community suicide prevention programs (Kato & Okada, 2019; Okada et al., 2020)—are correlated with reductions in regional suicide rates. Specific programs studied in previous research include minimum wages (Kaufman, Salas-Hernández et al., 2020; Kim et al., 2024), earned income tax credit (Dow et al., 2020), unemployment benefits (Antonakakis & Collins, 2015; Cylus et al., 2014; Kaufman, Livingstone, et al., 2020; Norström & Grönqvist, 2015), and employment protection legislation (Antonakakis & Collins, 2015; Kim & Cho, 2017). Additionally, personalized consultation support has demonstrated positive effects on mental health (McGrath et al., 2021), but evidence on suicide prevention remains limited, except in experimental studies (Barnes et al., 2018; 10.13039/100026819Jackson et al., 2022).

Whether personalized consultation on economic difficulties is associated with suicide prevention remains unclear. Distinguishing the effects of actual welfare programs from events that occur simultaneously, such as decreasing unemployment rates or long-term trends, on suicide rates remains a challenge. Further, comparing the contributions of specific program components is necessary to determine the optimal policy composition for achieving suicide prevention goals.

Japan's “Supporting the Self-Reliance of Persons in Need” program offers a unique opportunity to address this research gap. In recent years, social and economic changes have increased poverty in Japan. The society is facing a world top proportion of elderly population (Chiba, 2024), and a low birth rate of 1.2 compared to the world average of 2.2 (World Bank Group, 2023), which is intensifying the fiscal burden on the working-age population as the primary contributors to pension and tax revenues. According to Ministry of Health, Labour and Welfare (MHLW), the poverty measured by the number of recipients of public assistance rose consistently for 19 years before 2014 (MHLW, 2021a). The proportion of non-fulltime workers rose from 19.1 % of the labor force in 1989 to 36.7 % in 2013 (MHLW, 2024a). The increasing workforce receiving low salaries under unstable employment leaves the problem of unemployment and losing shelter. On the other hand, the public social spending in Japan composed 24.9 % of GDP in 2022, which is in the upper-middle tier among the OECD countries(OECD, 2024). This fiscal pressure has prompted welfare policy reforms aimed at reducing the spending on public assistance as well as improving the quality of services. Previous welfare programs, which were designed separately for older adults, people with disabilities, and children, were inadequate for addressing the complex situations faced by vulnerable populations (Kaburaki, 2020). The government intended to support the people in need with a second layer of safety net between the first net of the public insurance program and the third net of public assistance. When the welfare policies come into practice, the municipal governments have part of the autonomy to decide whether to apply for the pilot program, and the subprogram's contents. The local autonomy in Japan offers the opportunity to design a study leveraging diversity in municipal policy operations. In conclusion, self-reliance support in Japan provides an opportunity to validate a strategy for ensuring welfare provision under conditions of fiscal austerity despite substantial welfare expenditures.

**Fig. 1:**
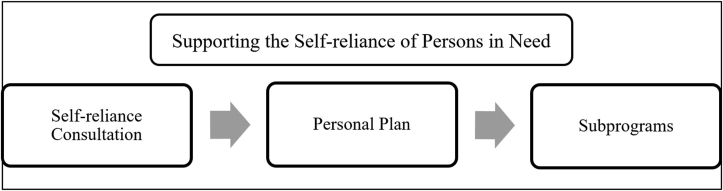
Processes of the “supporting the self-reliance of persons in need”.

In 2013, a partial amendment to the Public Assistance Act and Self-reliance Support Bill was passed to establish comprehensive self-reliance support for people in need and review the Japanese public assistance system. The legalization of Self-reliance Support clarified the duties to support the persons in need, which include the consultation program and subprograms provided by local governments, and the budget provided by the national government. The national government covers 3/4 to 1/2 budgets for programs under the “Supporting the Self-Reliance of Persons in Need” program. In 2013, the program was launched as a pilot in cities. In the pilot stage, there was one mandatory program, “self-reliance consultations,” and 4 subprograms were optional for local governments. After the national adoption, the policy added one more mandatory program, “residence security benefits,” and several subprograms. Fig. 1 provides a simplified explanation of the processes facilitated by the self-reliance support program. The mandatory consultation program is open to all who perceive themselves or others as having economic or self-reliance issues. It serves as an entry point for support, during which the support staff assess the difficulties faced by applicants and evaluate their motivation for self-reliance through employment and socialization. Based on the consultations, the staff develop personal plans for those needing further assistance by integrating locally available subprograms with existing welfare programs (MHLW, 2013a). In accordance with the applicants’ personal plans, they are guided to subprograms offering temporary benefits, employment support, and household finance support. The method part of the subprogram analysis provides detailed descriptions of each subprogram.

The pilot programs were initially launched in 47 cities in 2013 (MHLW, 2013b), expanded to 213 cities in 2014 (MHLW, 2014a), and finally implemented in the remaining 602 cities having regional welfare offices in April 2015, as mentioned in the Self-reliance Support for the Needy Act (MHLW, 2013c). Fig. A.1 in the Appendix illustrates expanding the “Supporting the Self-Reliance of Persons in Need” program in pilot cities. Following the national adoption of self-reliance support, the “Residence security benefits” program was added as a new mandatory program, and the optional programs were expanded in number and content. Since its national adoption in 2015, the number of applications for eight subprograms has been recorded by local governments.

The self-reliance support program differs from former welfare policies in two key aspects. First, it emphasizes the prevention of isolation through consultations and services to support employment and one's return to social life, unlike existing welfare policies, such as public assistance, which primarily offer tangible benefits, material assistance, and services. Second, a notable feature is that it provides support without rejecting applicants and is available to everyone, regardless of disability claims, unemployment, age, or nationality. Its goal is to create an early safety net for individuals not qualifying for public assistance or existing welfare programs before the situation worsens and the support methods are limited (Shinohara, 2022). Given the nature of the self-reliance support program, the government proposes collaboration with current suicide prevention policies (MHLW, 2023a).

Suicide remains a critical issue in Japan, which has consistently ranked first or second in suicide rates among G7 nations, an intergovernmental forum of seven major advanced countries, in the past decade (World Health Organization, 2021). In 2023, suicide accounted for 21,837 deaths in Japan (MHLW, 2024b), making it the leading cause of death for individuals aged 10−39 and the second or third leading cause for those aged 40−54 (MHLW, 2021b). Although there was a decade-long decline in suicide rates per 100000 population from 25.7 in 2009 to 15.9 in 2019, a new upward trend emerged with the onset of the COVID-19 pandemic in 2020 (MHLW, 2024b). Analyzing the factors that are related to the decrease in suicide rates during the 2010s will provide valuable insights for addressing the current rise.

This study hypothesizes that the introduction of the “Supporting the Self-Reliance of Persons in Need” program is linked to suicide reduction in Japan. By leveraging variations in the timing of policy implementation, the study examines the association of self-reliance support with monthly suicide rates at the city level, disaggregated by gender and age. Using difference-in-differences (DiD) analysis, this study compares cities that implemented pilot programs in 2013 and 2014 with those that did so in 2015. Additionally, it evaluates the association between the annual number of subprogram applications and annual suicide rates.

## 2 Methods

### 2.1 Analyses of the self-reliance support pilot program

#### 2.1.1 Data

The first analysis compared the monthly suicide rates in cities with and without exposure to the self-reliance pilot program before and after its introduction. The sample includes all cities and 23 special wards in Tokyo, accounting for 815 regions. Japan has a total of 1741 municipalities, yet the self-reliance support program is available only in 815 cities and 47 towns or villages having at least one welfare office. Having a welfare office is mandatory for cities and prefectures administering services under welfare laws, such as the Public Assistance Act and the Child, Aged, Disabled Welfare Act. To ensure comparability among cities, this study excluded 47 towns or villages with welfare offices that could operate self-reliance support. It compared cities that introduced the pilot program early with those that implemented it later nationwide. The MHLW provides data on the timing of the introduction (MHLW, 2013b; MHLW, 2014a). Table 1 outlines the program rollout: 47 cities started in 2013, 213 in 2014, and the remaining 602 in April 2015. The study period spans from January 2009 to December 2015, based on policy introduction timing and availability of city-level suicide data.

**Table 1:** Timing of the introduction of self-reliance support in pilot cities.

Date	New pilot cities	Total pilot cities	Non-pilot cities
	0	0	815
January 2013	9	9	806
April 2013	12	21	794
May 2013	1	22	793
July 2013	2	24	791
August 2013	1	25	790
September 2013	1	26	789
October 2013	18	44	771
November 2013	2	46	769
December 2013	1	47	768
January 2014	3	50	765
April 2014	68	118	697
May 2014	4	122	693
June 2014	21	143	672
July 2014	11	154	661
August 2014	2	156	659
September 2014	1	157	658
October 2014	48	205	610
November 2014	5	210	605
December 2014	3	213	602

#### 2.1.2 Analytical strategy

To assess the self-reliance support program's association with suicide rates in pilot cities, a DiD design was employed by leveraging variations in the timing of the program's introduction. The main estimation was based on the following model:

(1) $$Y_{cym}=\beta_{0}+\beta_{1}{Support}_{cym}+\beta_{2}X_{cy}+\varphi_{c}+\mu_{ym}+\varepsilon_{cym}$$

The outcome variable, $Y_{cym}$, is the suicide rate for the specific demographic group in city *c* in year *y* and month *m*. The study period spans from January 2009 to December 2015. Suicide rates are calculated by dividing suicide counts by the population and multiplying by 100,000 for each gender-age-city-time cell.^1^ The population is stratified by gender and four age groups: total population, age under 29, age 30−59 years, and age above 60 years. Suicide counts for each cell have been available from the MHLW at the municipal level since January 2009 (MHLW, 2024c). The MHLW compiles the data based on the original data of suicide statistics provided by the National Police Agency (Japan Suicide Countermeasures Promotion Center, 2025). The MHLW provides suicide data by date of execution or discovery, and place by residence or discovery. I chose the suicide record of execution date and the place of residence to measure the policy association with cancelling the decision. Different from another suicide data source, “Vital Statistics,” which only collects data from Japanese residents, the data from the National Police Agency includes all Japanese and foreign residents (MHLW, 2025). It is consistent with the policy range for all residents regardless of nationality. However, the municipality's monthly data is provisional, which might have a small difference from the actual incident. Population data by demographic groups are sourced annually from the population count, vital events, and households from the Basic Resident Registration (Ministry of Internal Affairs and Communications, 2024).

The main treatment variable, ${Support}_{cym}$, is a dummy variable that equals 1 if the pilot self-reliance support program has started in city *c* in year *y*, month *m*, and later, and 0 otherwise. Socio-economic confounders, $X_{cy}$, capture the time-varying characteristics of city *c* in year *y*, selected based on prior studies (Kuroki, 2010; Mizushima & Noguchi, 2021). These include the natural logarithm of the population by demographic groups, the natural logarithm of average taxable income, proportions of the population aged 15 or younger and 65 or older, unemployment rates, birth rates, marriage and divorce rates, and the natural logarithm of per capita expenditures on social welfare and public assistance. Data that were surveyed every 5 years and missing data were linearly interpolated. The city and year-by-month fixed effects are denoted by $\varphi_{c}$ and $\mu_{ym}$, respectively. Standard errors are clustered at the city level to account for city-specific heteroskedasticity and autocorrelation, and estimations are weighted by city population.

For the robustness test, I first employed a Poisson pseudo–maximum-likelihood estimator to address the high incidence of zero suicide counts over the study period (Correia et al., 2020; Tanaka & Okamoto, 2021) (Table A.1). I also aggregated the data into quarterly or semiannual intervals—rather than using monthly counts—to further mitigate the influence of zeros. To account for heterogeneous treatment effects across intervention groups and over time, I applied both the two-stage difference-in-differences estimator (Gardner, 2022) (Table A.2) and a staggered DiD estimator (Callaway & Sant’Anna, 2021), compared each with the baseline model. To isolate the self-reliance support from contemporary suicide prevention policies, I controlled for prefecture-level allocations from the Emergency Fund to Enhance Community-Based Suicide Countermeasures. In heterogeneity analyses, I examined how the correlation varies by city characteristics, specifically, nonprofit organization density and whether the population exceeds 200,000. Finally, I conducted placebo tests on alternative causes of death presumed to be unaffected by the intervention, thereby confirming the specificity of the findings.

A valid DiD analysis relies on the parallel trends assumption, which means that without the policy intervention, suicide rates in pilot and non-pilot cities would follow similar trends over time. To check this assumption indirectly, I conducted event-study analyses. These analyses track suicide rates before and after policy introduction to detect changes. If the assumption holds, there should be no significant differences in suicide rates between pilot and non-pilot cities before the policy's implementation. One concern is that suicide rates may not respond immediately to policy introduction because stress-related suicides typically develop gradually. Another issue is that monthly suicide counts for specific groups (by gender, age, and city) can be quite low, causing fluctuations around the policy introduction period. To address this, I used six-month intervals instead of monthly data in the event studies.

(2) $$Y_{cym}=\beta_{0}+\sum_{k=-48,\neq -6}^{30}\beta_{1,k}1\left(t-t_{c}^{*}=k\right)+{\beta_{2}X}_{cy}+\varphi_{c}+\mu_{ym}+\varepsilon_{cym}$$

The event-time dummies, $1\left(t-t_{c}^{*}=k\right)$, equal 1 when the observation year *y* and month *m* are within *k* = −48, −42, −36 …, and 0, 6, 12 …, 30 months, respectively, from $t_{c}^{*}$, the year and month when the pilot city *c* introduced the self-reliance support program. The −48 is the earliest window that includes observations from all cities because the analysis starts from 2009, and the policy adoption starts from 2013. *K* = −6 is omitted to normalize the estimation of $\beta_{1,k}$ to 0 in the time unit before the program adoption. Estimates of $\beta_{1,k}$ for the pretreatment period, when *k* < 0, are expected to be near 0, serving as a check for the parallel trends assumption. If self-reliance support reduces the suicide rates, $\beta_{1,k}$ for *k* = 0 shifts discontinuously to negative and remains negative for *k* > 0. To indirectly assess the validity of the parallel trends assumption over a longer pretreatment period, I performed event study analyses using annual data from 2003 to 2015, as shown in Figure A.2, based on suicide statistics from the “Vital Statistics”.

### 2.2 Analyses of optional subprograms

#### 2.2.1 Data

The second analysis aims to examine which types of initiatives under the self-reliance support program were related to the changes in suicide rates. This study analyzed the number of new applications and eight subprograms, using records categorized by city and fiscal year (April to March of the following year). The subprograms can be classified into three categories: temporary benefits, employment support, and household finance support. Table 2 provides detailed descriptions of each subprogram. The self-reliance support program, along with the “self-reliance consultation” and “residence security benefits” subprograms, have become mandatory in all cities since 2015. Local governments have the discretion to implement optional subprograms, although broader implementation is strongly encouraged.

**Table 2:** Contents of the optional programs of supporting the self-reliance of persons in need.

Program group	Program name	Contents
Temporary benefits	Residence security benefits	The amount equivalent to rent will be provided. For those who have lost or are likely to lose their housing owing to separation from employment, etc., an amount equivalent to rent will be provided for a certain period on the condition that they engage in activities to find employment. After establishing a residence that will serve as a foundation for their livelihood, the program will support them in finding employment. This program is available to those meeting certain asset, income, and other requirements.
	Temporary living support program	Food, clothing, and shelter are provided to those without housing. A place to stay, food, and clothing for a certain period are also provided to those who do not have a residence or are in unstable housing situations, such as Internet cafes. They are also provided with self-support assistance, such as employment support, to prepare for life after leaving the facility.
Employment support	Employment preparation support program (since the pilot period)	The first step toward society and employment. For those who have difficulty finding employment immediately because they are anxious about social relations or have difficulty communicating with others, support and work opportunities are provided while developing basic skills for general employment in accordance with a program that lasts from 6 months to 1 year.
	Employment training programs (since the pilot period)	Providing work opportunities through flexible work styles. Those who have difficulty finding mainstream employment after the employment preparation program are also equipped with employment training programs (so-called “intermediate employment”) that provide medium- to long-term support toward general employment based on individualized employment (i.e., self-reliance support while providing work opportunities suited to the individual).
	Employment support through consultation support services for self-reliance	For those who can find mainstream employment after a period of individual support, advice, and guidance on employment, individual job searches, accompaniment to Hello Work (the Public Employment Security Office), support with job placement, etc., are provided by employment support workers assigned to disability support services.
	Employment promotion for public assistance recipients, etc.	For those having better preparation for employment than those in the above category, but are expected to be employed with individualized support. Through cooperation between the Labor Bureau, Hello Work, and local public entities, this project aims to promote the self-reliance of public assistance recipients through employment by establishing a nationwide one-stop employment support system and providing employment support through the cooperation of related organizations.
Household finance support	Household Finance Improvement Support program (since the pilot period)	Advice on rebuilding household finances. To “visualize” the household financial situation and understand the underlying issues, as well as to help borrowers manage their household finances on their own, the early restoration of livelihood is supported by preparing a support plan according to the situation, providing counseling and support, connecting borrowers to relevant institutions, and, if necessary, mediating loans. This service is available to those who meet certain asset and income requirements.
	Loans from livelihood and welfare funds	This program aims to promote the economic independence and motivation of low-income individuals, those with disabilities, and older adults, as well as to foster home welfare and social participation by providing loans and necessary counseling and support to enable them to lead stable lives.

Note: The “Supporting the Self-Reliance of Persons in Need” also provides other optional programs, such as support for children's livelihood and education (since the pilot period). However, application case data are unavailable for optional programs other than the ones listed above.

Data on new applications and subprograms from the MHLW cover application records from 2015 to 2021. While the pilot cities began part of the subprograms in 2013 and 2014, application records are available only from 2015. In the non-pilot cities, no subprograms were in place before 2015. Since then, the subprograms have been gradually introduced in non-pilot cities. The most popular subprogram was the “Household Finance Improvement Support Program,” which operated in 83 % of cities. The “Temporary living support program” was adopted in only 43 % of cities (MHLW, 2023b). This study analyzed in long (from 2009 to 2021) and short (from 2015 to 2021) periods. In the long-period analyses, the sample is restricted to non-pilot cities to avoid missing data for subprograms in pilot cities in 2013 and 2014. After excluding two newly created cities that do not have complete data, the sample size of the long-period subprogram analyses comprises 600 non-pilot cities. In the short-period analysis, I included all 815 cities because the subprogram application data are available for all cities since 2015.

#### 2.2.2 Analytical strategy

Using new application cases per 100,000 population for the self-reliance support program, DiD analyses are conducted with continuous treatment variables. Simultaneously, subprograms with application records are included in the model, as described below:

$$Y_{cy}=\beta_{1\;}{newconsultation}_{cy}+\sum_{k=1}^{8}\beta_{2,k}{subprogramK}_{cy}+\beta_{3\;}{newconsultation}_{cy}\times After2020$$

(3) $$+\sum_{k=1}^{8}\beta_{4,k}{subprogramK}_{cy}\times After2020+{\beta_{3}X}_{cy}+\varphi_{c}+\mu_{y}+\varepsilon_{cy}$$

The dependent variable, $Y_{cy}$, is the suicide rate for a specific demographic group in city *c* during fiscal year *y*. Suicide rates per fiscal year are calculated from aggregate monthly suicide counts and annual population. To distinguish the consultation and actual support, the treatment variable ${newconsultation}_{cy}$, which represents new application cases for the self-reliance support program per 100,000 population, is added. Additionally, $sub{programK}_{cy}$ represents the application cases per 100,000 population for each subprogram in city *c* during fiscal year *y*. Since not all applicants are provided with subprograms after consultation, and one applicant might receive support from several subprograms, the variable ${newconsultation}_{cy}$ is hardly equal to the sum of ${subprogramK}_{cy}\text{.}$ Notably, new applications for self-reliance support surged after the COVID-19 pandemic as compensation for economic damages. New applications rose from 16.2 per 100,000 population in 2019 to 51.4 in 2020 and remained high at 36.6 in 2021. To capture the association in both normal and pandemic years, interaction terms of the treatment variables were used with the dummy variable After2020, which equaled 1 when the year was 2020 or later and 0 otherwise. The analysis chose 2 periods from 2009 to 2021 for the non-pilot city sample, or from 2015 to 2021 for the complete 815 cities, according to the availability of suicide data and subprogram application records.

## 3 Results

### 3.1 Analyses of the self-reliance support pilot program

#### 3.1.1 Descriptive statistical analysis

**Fig. 2:**
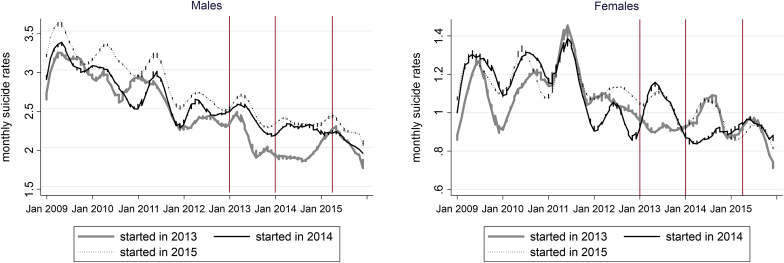
Trends in monthly suicide rates stratified by self-reliance support start years and gender, from January 2009 to December 2015.

Fig. 2 illustrates the trends in monthly suicide rates in cities from 2009 to 2015. The suicide rates were higher in the non-pilot cities that implemented self-reliance support in April 2015, as compared with those that adopted it earlier in 2013 or 2014. This may indicate that the cities with better welfare systems are more likely to adopt new policies earlier and also have stronger suicide prevention measures. This trend was more pronounced among males, who had higher suicide rates than females. Between early and late-adopting cities, female suicide rates showed little difference. However, both genders showed reductions in suicide rates in the year following implementation, when monthly suicide rates before and after the policy introduction were compared with those in non-pilot cities.

Descriptive statistics for control variables and the outcomes are displayed in Table 3. City characteristics were generally similar in value among early and late-adopting cities. The early-adopting cities tend to have a large population scale, a smaller proportion of older adults, and higher marriage rates. Besides, early-adopting cities had higher per capita expenditure on social welfare and public assistance, which was consistent with lower average suicide rates. To assess the baseline comparability between early- and late-adopting cities, I presented the result of a *t*-test for difference comparing the early-adopting cities with the late-adopting cities in 2015. The differences of most covariates and outcomes were statistically significant, indicating the need for careful identification strategies comparing treatment and control groups.

**Table 3:** Descriptive statistics.

	Start in 2015	Start in 2014		Start in 2013	
Panel A. City Characteristics	Mean	Mean	Difference	Mean	Difference
Log population	11.2	11.7	−0.478∗∗∗	12.2	−1.013∗∗∗
Log average taxable income	8.0	8.0	−0.028∗∗∗	8.0	−0.070∗∗∗
% of the population aged 15 or younger	12.8	13.0	−0.263∗∗∗	12.8	−0.031
% of the population aged 65 or older	26.9	25.9	0.985∗∗∗	25.3	1.611∗∗∗
Unemployment rate	5.6	5.4	0.119∗∗	5.6	−0.053
Birth rate	7.6	8.0	−0.363∗∗∗	7.9	−0.292∗∗∗
Marriage rate	4.7	4.9	−0.221∗∗∗	5.0	−0.286∗∗∗
Divorce rate	1.8	1.8	−0.018	1.8	−0.049∗∗
Log per capita expenditure on social welfare	35.3	35.9	−0.597∗	35.8	−0.445
Log per capita expenditure on public assistance	2.7	2.9	−0.193∗∗∗	3.1	−0.398∗∗∗
Number of observations	602	166		47	
Panel B. Outcomes	Mean	Mean	Difference	Mean	Difference
Monthly suicide rates: total	1.9	1.8	0.109∗∗∗	1.7	0.180∗∗∗
Monthly suicide rates: male	2.7	2.5	0.191∗∗∗	2.4	0.312∗∗∗
Monthly suicide rates: female	1.1	1.1	0.029	1.0	0.052

Notes: Annually measured city characteristics were derived from the Portal Site of Official Statistics of Japan (e-Stat). Data that were surveyed every 5 years and missing data were linearly interpolated. 3 city characteristics “% of the population aged 15 or younger”, “% of the population aged 65 or older”, and “Unemployment rate” were linear interpolation of five-year survey. The rest of the characteristics had yearly data from 2009 to 2020, while the “Birth rate”, “Marriage rate”, and “Divorce rate” still had data for 2021.

#### 3.1.2 Baseline regression results

Table 4 shows the estimated association of introducing self-reliance support in pilot cities with monthly suicide rates, separated by gender and age groups. A more pronounced negative association in male monthly suicide rates at 0.066 (95 % Confidence Intervals [CI] = −0.138 to 0.007) was reported in the Model with all covariates in column 1 following the introduction of self-reliance support in pilot cities. However, the coefficients were smaller for females at 0.041 (95 % CI = −0.080–0.000) in column 2. When examining age groups, the association was stronger for individuals aged 30−59 years, particularly among middle-aged men, who showed the largest of 0.122 (95 % CI = −0.241 to 0.003) in column 5. Middle-aged women showed a smaller coefficient of 0.068 (95 % CI = −0.144 to −0.002) in column 6. No significant association was detected for other age groups, except for females under 29 years, who estimated a association of 0.063 (95 % CI = −0.124 to −0.004) in column 4. I reported the Fisher tests for t-statistics of the coefficients. Small *p*-values for Fisher tests under the significant coefficients suggested the small possibility of observing t-statistics as large as the real value. It provides evidence that the DiD estimates are not merely a random artefact of the data. The Robustness tests used the Poisson pseudo-maximum-likelihood estimator, as shown in Table A.1, confirmed the main finding that introducing self-reliance support in pilot cities is related to the reduction in monthly suicide rates, particularly among middle-aged and younger females.

**Table 4:** Analyses on monthly suicide rates after introducing self-reliance support in pilot cities, by gender and age.

Age	All		≤29		30–59		≥60	
Gender	M	F	M	F	M	F	M	F
	(1)	(2)	(3)	(4)	(5)	(6)	(7)	(8)

Support (without covariates)	−0.055	−0.038∗	−0.049	−0.070∗∗	−0.085	−0.073∗∗	−0.009	0.026
	(0.042)	(0.022)	(0.049)	(0.032)	(0.065)	(0.036)	(0.065)	(0.038)
								
Support (with nonlinearly interpolated covariates)	−0.064	−0.040∗	−0.041	−0.061∗∗	−0.120∗	−0.067∗	−0.003	0.011
	(0.040)	(0.021)	(0.046)	(0.030)	(0.067)	(0.036)	(0.065)	(0.038)
								
Support (with all covariates)	−0.066∗ (0.037)	−0.041∗∗ (0.020)	−0.042 (0.046)	−0.063∗∗ (0.030)	−0.122∗∗ (0.061)	−0.068∗ (0.036)	−0.005 (0.065)	0.010 (0.039)
Fisher's P-value of the analyses with all covariates	0.071	0.063	0.376	0.047	0.050	0.071	0.964	0.778
Fixed effects	✓	✓	✓	✓	✓	✓	✓	✓

Notes: Robust standard errors are shown within parentheses. ∗∗∗p < 0.01, ∗∗p < 0.05, ∗p < 0.1. Difference-in-differences analysis included time-varying city characteristics and fixed effects for city, year, and month, estimated separately by gender and age groups. Each model yielded 68460 observations from 815 cities, 7 years, and monthly. The study period was from 2009 to 2015. Standard errors were clustered at the city level.

#### 3.1.3 Event study results

**Fig. 3:**
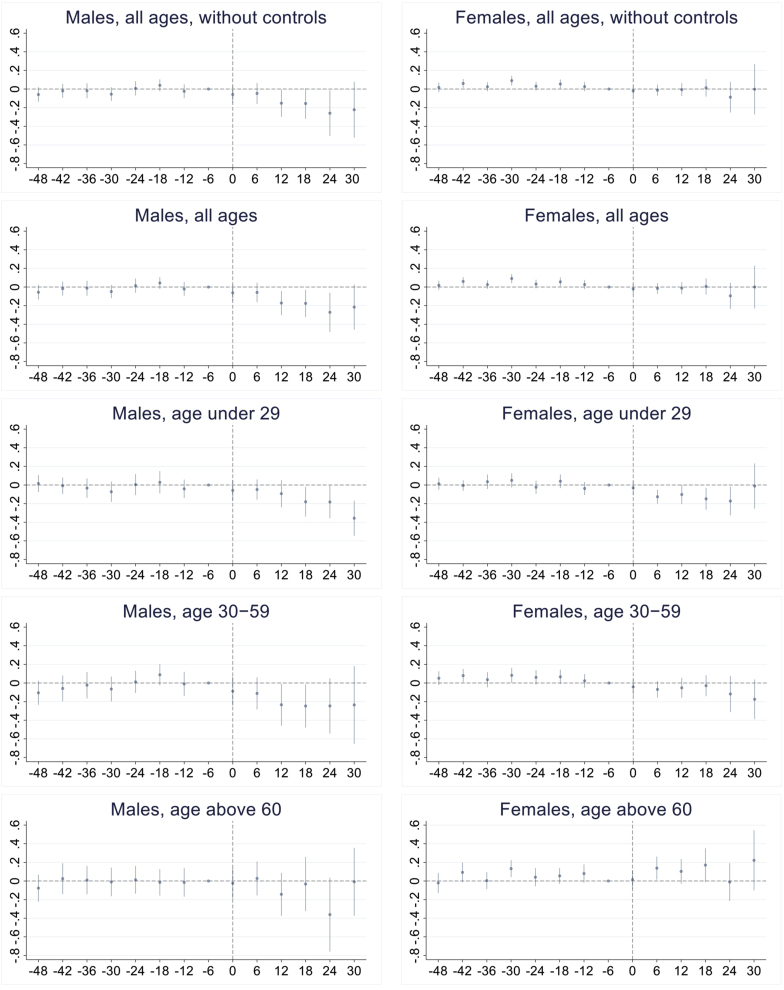
Event studies by gender and age groups.

Fig. 3 shows the results of event studies by gender and age groups, estimated coefficients with 95 % CI for each time unit. The period of the event studies ranged from 4 years before the intervention to 2.5 years after it. The event study range was decided according to the stable and fixed observations for each window to avoid the extreme estimation for far windows without sufficient observations. The parallel trends assumption was indirectly supported if pre-intervention coefficients are near zero. I conducted joint F-tests of the null hypothesis that all pre-treatment coefficients were equal to zero for the total, male, and female populations, with p-values of 0.305, 0.167, and 0.025, respectively. The null hypothesis couldn't be rejected for the total and male populations. Therefore, it possibly supported the parallel trends assumption for these groups. However, the existence of pretreatment differences draws caution for the results of the female groups. Following the process in the basic analyses that included the control variables first, I reported the results without control variables for the male and female populations. The results were similar whether included the controls or not. For the male population, coefficients remained close to zero before the intervention and decreased afterward. For the female population, while pre-intervention coefficients diverged from zero, post-intervention coefficients did not show a clear decline. The results of the event studies were consistent with those of the DiD models, indicating a stronger association for males than for females. Event studies for other age groups also confirmed the DiD models, that sudden drops in the coefficients occurred after interventions for the middle-aged and females under 29 years. Conversely, the event study for males under 29 years captured a negative association not observed in the DiD model.

#### 3.1.4 Robustness tests: quarterly or half-yearly time intervals

Table 5 reports the pilot program's association with suicide rates when data were aggregated to the quarterly or half-yearly levels, compared results from DiD models with those from the CS-DiD method (Callaway & Sant’Anna, 2021). Aggregating data to quarterly or half-yearly intervals helped address inherent limitations in the monthly data at the municipality level. Specifically, the number of suicide deaths in some municipalities was small, causing the monthly suicide rate to be unstable and contain zeros. This instability became even more pronounced in subgroup analyses by sex and age groups. Thus, aggregating the data to quarterly or half-yearly windows may yield more stable estimates.

**Table 5:** Robustness tests using quarterly or half-yearly time intervals, and staggered DID.

	DID: Quarter		DID: Half year		CSDID: Quarter		CSDID: Half year	
Gender	M	F	M	F	M	F	M	F
	(1)	(2)	(3)	(4)	(5)	(6)	(7)	(8)

Support	−0.231∗	−0.157∗∗	−0.426∗	−0.466∗∗∗	−0.362	0.001	−0.768	−0.241
	(0.121)	(0.065)	(0.247)	(0.128)	(0.341)	(0.181)	(0.479)	(0.313)
Average ATT before treatment					−0.081∗	0.024	−0.054	0.048
					(0.043)	(0.021)	(0.117)	(0.060)
Average ATT after treatment					−0.957∗∗	−0.024	−1.429∗∗	−0.305
					(0.439)	(0.269)	(0.590)	(0.402)
Observations	20,375	20,375	9780	9780	20,375	20,375	9780	9780
Covariates	✓	✓	✓	✓	✓	✓	✓	✓
Fixed effects	✓	✓	✓	✓	✓	✓	✓	✓

Notes: Robust standard errors are shown within parentheses. ∗∗∗p < 0.01, ∗∗p < 0.05, ∗p < 0.1. Difference-in-differences analysis included time-varying city characteristics and fixed effects for city, and quarterly or half-yearly time unit, estimated separately by gender groups. The study period was from January 2009 to March 2015 for the quarterly analyses, and from January 2009 to December 2014 for half-year analyses to avoid the policy adoption in non-polit cities. Standard errors were clustered at the city level.

Across both quarterly and half-yearly aggregations, the program's association with suicide rates remained negative and statistically significant, consistent with the monthly findings. In the quarterly data, the DiD estimates indicated a significant negative association with suicide rates following the program, with a magnitude roughly equivalent to the monthly effect accumulated for a quarter year. Similarly, the CS-DiD estimates for quarterly or half-yearly data also showed a significant negative ATT after treatment in the male group. The consistency of results across different time aggregations underscores the robustness of the program's negative association with suicide rates. Comparisons between DiD and CS-DiD showed that while point estimates were closely aligned in direction, the CS-DiD estimated ATT in the first line tended to be more conservative with wider confidence intervals.

#### 3.1.5 Robustness tests: control contemporary suicide prevention policies

The estimated associations for the self-reliance support might just reflect the effect of the contemporary policies that affected suicide rates. To isolate the association of self-reliance support, I reviewed the government measures to prevent suicide during the study period and included them in the estimation. The main related policies include revision of the General Principles of Suicide Prevention Policy in 2012, Basic Law on Measures to Prevent Damage to Health Due to Alcohol in 2013, and the Act Promoting Measures to Prevent Death and Injury from Overwork in 2014 (MHLW, 2016; Okamura et al., 2021; Takeshima et al., 2015). They were not included in the estimation due to either the national adoption or the execution date later than the study period. One worth noting policy was the Emergency Fund to Enhance Community-Based Suicide Countermeasure lasted from 2009 to 2014 (MHLW, 2014b). It was a prefecture-level policy that allocated 2 kinds of suicide prevention funds according to the application by prefecture government and their administering municipal governments. The start of the Emergency Fund coincided with the start of suicide decrease in 2009. Literatures also support the association between the policy and suicide decrease (Kato & Okada, 2019; Okada et al., 2020). I included the Emergency Fund per 100000 population by prefecture-year in the basic model in 2 forms. One was the aggregated funds for both the prefecture and municipality levels to represent the actual amount of funds received by prefectures. The other was the municipality-level funds with available data in prefecture-year panels to represent various efforts by municipal governments in suicide prevention. The emergency fund stopped in 2015 and was replaced by regular funds without panel data on the fund amount by prefecture. Therefore, I compared the model with and without controlling for the found before January 2015. Table 6 displays the results when adding the 2 forms of Emergency Funds gradually into the model. The coefficients of self-reliance support did not change much when controlled for the Emergency Fund. The coefficients of different forms of the Emergency Fund were all close to zero. The prefecture panel data of the Emergency Fund does not support that the association of self-reliance support comes from the reflection of the main suicide prevention policy during the study period.

**Table 6:** Analyses on monthly suicide rates after introducing self-reliance support in pilot cities, controlling for the Emergency Fund to Enhance Community-Based Suicide Countermeasure.

Gender	M	F	M	F	M	F	M	F
	(1)	(2)	(3)	(4)	(5)	(6)	(7)	(8)

Support	−0.066	−0.057∗∗	−0.068	−0.057∗∗	−0.064	−0.057∗∗	−0.065	−0.057∗∗
	(0.043)	(0.024)	(0.043)	(0.024)	(0.042)	(0.024)	(0.042)	(0.024)
Total budget rate			0.000∗	−0.000			0.000	−0.000
			(0.000)	(0.000)			(0.000)	(0.000)
Municipal budget rate					0.000∗∗	−0.000	0.000	−0.000
					(0.000)	(0.000)	(0.000)	(0.000)
Observations	58,680	58,680	58,680	58,680	58,680	58,680	58,680	58,680
Covariates	✓	✓	✓	✓	✓	✓	✓	✓
Fixed effects	✓	✓	✓	✓	✓	✓	✓	✓

Notes: Robust standard errors are shown within parentheses. ∗∗∗p < 0.01, ∗∗p < 0.05, ∗p < 0.1. Difference-in-differences analysis included time-varying city characteristics and fixed effects for city, year, and month, estimated separately by gender. Each model yielded 58680 observations from 815 cities, 6 years, and monthly. The study period was from 2009 to 2014. Standard errors were clustered at the city level.

#### 3.1.6 Heterogeneity analysis

Table 7 explores heterogeneity in the program's estimated associations by two key contextual factors: the density of non-profit organizations (NPOs), used as a proxy for the strength of welfare foundation and social capital; and the population size, which reflects urban status and administrative capacity. I divided the cities into two groups with high or low values for these factors. I chose NPO density as a key variable based on both theoretical considerations of social capital and practical insights into local government collaboration with volunteer organizations. Theoretically, a high density of NPOs is indicative of rich community social capital (Saxton & Benson, 2005), which has been linked to lower suicide rates (Smith & Kawachi, 2014). Practically, in implementing the self-reliance support program, some local governments actively sought assistance from existing NPOs that provided services such as food and shelter (Shinohara, 2022). Municipalities with a foundation of collaboration with NPOs offering services aligned with the program were more likely to apply to become pilot cities, as doing so entailed fewer additional administrative burdens. Therefore, I analyzed the heterogeneous results of the pilot program considering the differences in NPO density. The NPO density was calculated from the number of all kinds of NPO per 100000 population in 2013 (Cabinet Office, 2025). I used another moderating factor of the city population compared with 200000. It is not only a key factor of city scale, but also a necessary condition to apply for the core city in Japan. There are 82 core cities and ordinance-designated cities within the 815 cities, which share the administrative authority with the prefecture government to improve access to services.

**Table 7:** Heterogeneity analysis grouped by NPO density and Population compared with 200000.

	NPO Density				Population in 2012 compared with 200000			
Group	High	Low	High	Low	More	Less	More	Less

Gender	M	M	F	F	M	M	F	F

	(1)	(2)	(3)	(4)	(5)	(6)	(7)	(8)

Support	−0.110∗∗	−0.012	−0.075∗∗∗	−0.001	−0.070∗	−0.035	−0.039	−0.052
	(0.044)	(0.057)	(0.025)	(0.033)	(0.041)	(0.074)	(0.025)	(0.043)
Observations	34,068	34,392	34,068	34,392	10,920	57,540	10,920	57,540
Covariates	✓	✓	✓	✓	✓	✓	✓	✓
Fixed effects	✓	✓	✓	✓	✓	✓	✓	✓

Notes: Robust standard errors are shown within parentheses. ∗∗∗p < 0.01, ∗∗p < 0.05, ∗p < 0.1. Difference-in-differences analysis included time-varying city characteristics and fixed effects for city, year, and month, estimated separately by gender. Each model yielded observations from High + Less = 815 cities, 7 years, and monthly. The study period was from 2009 to 2015. Standard errors were clustered at the city level.

The results suggest that the coefficients of the self-reliance support program varied according to these city characteristics. In areas with a high NPO density, the program's association with suicide rates was particularly pronounced and statistically significant in both male and female groups. This implies that in communities where civil-society support networks were more abundant, the program was especially associated with a fast reduction in suicide rates, possibly because local NPOs complement the program's services or helped reach at-risk individuals more effectively. By contrast, in low NPO density areas, the program's coefficients were smaller and not statistically significant. This contrast highlights that the presence of supportive non-profit institutions may amplify the program's association. A similar pattern of heterogeneity was observed with respect to population size. In cities with larger populations and possibly administrative power, the coefficients on the program indicator were relatively large in magnitude and statistically significant for males. Conversely, in smaller cities, the estimated coefficients were smaller in magnitude, less precisely estimated, and statistically insignificant. One potential explanation is that larger municipalities may have more established administrative capacities and resources, enabling more effective and systematic implementation of the intervention. In summary, Table 7 indicates important heterogeneity: negative associations with suicide were found primarily in high-NPO-density and high-population cities. These findings imply that local social infrastructure and city scale may influence the result of the intervention.

#### 3.1.7 Placebo test

Table 8 reports results from placebo tests, where the outcomes are other causes of death that the program was not intended to associate with. I used the annual death rates by cities for 4 different causes of death, including all deaths except suicide, the top three causes of death, cancer, heart diseases, and senility (MHLW, 2023c). The death rates were calculated in the same way as the suicide rates, except that the number of deaths is annual. I used the annual version of equation one. Consistent with expectations, Table 8 shows no negative association with the top causes of death. For each placebo outcome, the estimated coefficients on the program were statistically indistinguishable from zero, or slightly positive for causes except suicide for females. The implication is that the program's introduction did not coincide with the tested specific non-suicide death causes. In sum, the placebo test demonstrates that the negative association with suicide is specific, which adds credibility to the claim that the results of the program are less likely to reflect an unobserved factor that would also affect other mortality.

**Table 8:** Placebo test with other causes of death.

Cause of death	Except suicide		Cancer		Heart diseases		Senility	
Gender	M	F	M	F	M	F	M	F

	(1)	(2)	(3)	(4)	(5)	(6)	(7)	(8)

Support	3.513	5.508∗	−0.401	0.971	−0.612	0.637	0.106	0.955
	(3.321)	(3.187)	(1.916)	(1.258)	(1.254)	(0.940)	(0.555)	(1.174)
Covariates	✓	✓	✓	✓	✓	✓	✓	✓
Fixed effects	✓	✓	✓	✓	✓	✓	✓	✓

Notes: Robust standard errors are shown within parentheses. ∗∗∗p < 0.01, ∗∗p < 0.05, ∗p < 0.1. Difference-in-differences analysis included time-varying city characteristics and fixed effects for city and year, estimated separately by gender groups. Each model yielded 5705 observations from 815 cities and 7 years. The study period was from 2009 to 2015. Standard errors were clustered at the city level.

### 3.2 Association of optional subprograms with suicide rates

Table 9 indicates that higher application rates for the employment training program were correlated with lower annual suicide rates at the city level. However, the application rates for new consultations during both normal and pandemic years showed no significant association, suggesting that merely increasing the number of self-reliance support applications may not meaningfully relate to suicide prevention. Analyses of the eight subprograms revealed that the employment training program exhibited the strongest correlation with reduced suicide rates in both normal and pandemic years, particularly among males. During normal years (2009–2019), one additional application per 100,000 population for employment training was associated with a reduction of 0.256 and 0.169 in the annual male and female suicide rates, respectively. In the pandemic years (2020–2021), the additional applications for employment training correlated with a marginal reduction of 0.359 in male suicide rates, corresponding to a total reduction of 0.615 in suicide rates. No significant associations were identified for subprograms in other categories, such as those providing temporary benefits or household finance support. When estimated in the short study period from 2015 to 2021, the negative correlation of the employment training program with suicide rates increased in magnitude in both normal years and pandemic years. No negative correlations were observed for other subprograms.

**Table 9:** The association of subprogram application rates with annual suicide rates.

	Non-pilot cities 2009–2021		All cities 2015–2021	
Gender	Males	Females	Males	Females
	(1)	(2)	(3)	(4)
Application rates				
New consultation	0.024	0.009	0.010	0.007
	(0.023)	(0.013)	(0.031)	(0.016)
Residence security benefits	−0.004	0.002	0.019	−0.001
	(0.031)	(0.016)	(0.031)	(0.021)
Temporary living support	0.036	−0.026	0.104∗	−0.022
	(0.039)	(0.016)	(0.059)	(0.035)
Employment preparation	−0.030	−0.002	0.019	−0.006
	(0.032)	(0.023)	(0.031)	(0.026)
Employment training	−0.256∗∗	−0.169∗∗	−0.449∗∗∗	−0.297∗∗∗
	(0.115)	(0.069)	(0.138)	(0.107)
Employment support through consultation	0.017	0.001	0.010	0.001
	(0.012)	(0.007)	(0.012)	(0.008)
Employment promotion for public assistance recipients, etc.	−0.008	−0.005	0.022	0.000
	(0.016)	(0.010)	(0.019)	(0.014)
Household finance improvement	0.005	0.002	−0.009	−0.003
	(0.011)	(0.005)	(0.011)	(0.006)
Loans from livelihood and welfare funds	−0.014	−0.005	0.017	−0.003
	(0.030)	(0.017)	(0.031)	(0.020)
Application rates × After 2020				
New consultation	−0.016	−0.008	−0.007	−0.007
	(0.025)	(0.014)	(0.031)	(0.015)
Residence security benefit	−0.002	−0.011	−0.023	−0.007
	(0.031)	(0.016)	(0.030)	(0.021)
Temporary living support	−0.059	0.005	−0.028	−0.005
	(0.044)	(0.030)	(0.043)	(0.030)
Employment preparation	0.050	−0.006	0.003	−0.006
	(0.032)	(0.024)	(0.031)	(0.027)
Employment training	−0.359∗	0.243	−0.530∗∗∗	0.128
	(0.194)	(0.224)	(0.205)	(0.248)
Employment support through consultation	−0.008	0.008	−0.002	0.007
	(0.012)	(0.007)	(0.012)	(0.008)
Employment promotion for public assistance recipients, etc.	0.016	0.001	−0.001	−0.001
	(0.019)	(0.012)	(0.019)	(0.014)
Household finance improvement	−0.003	0.002	0.008	0.005
	(0.011)	(0.006)	(0.011)	(0.006)
Loans from livelihood and welfare fund	0.011	0.004	−0.020	0.002
	(0.030)	(0.017)	(0.031)	(0.020)
Observations	7800	7800	4200	4200

Notes: Robust standard errors are shown within parentheses. ∗∗∗p < 0.01, ∗∗p < 0.05, ∗p < 0.1. New consultations and subprograms were measured by applications per 100000 population by fiscal year, from April to March of the following year. The annual suicide rates were calculated by fiscal year from monthly suicide counts and annual population. Fixed effects analysis included time-varying city characteristics, fixed effects for cities, and year. Standard errors were clustered at the city level.

## 4 Discussion

This study investigates “Supporting the Self-Reliance of Persons in Need”, a personalized economic support program in Japan, with city suicide rates. The introduction of self-reliance support is associated with an average reduction of 0.05 suicides per 100,000 population per month, representing a 2.69 % decrease in the average monthly suicide rate and equating to approximately 763 fewer yearly suicides based on the 2015 Japanese population. During the pilot period, the national suicide rates reduced from 21.8 per 100,000 population in 2012 to 18.9 in 2015. This policy has stronger estimates for males and middle-aged individuals. The association with reducing suicides is also observed in the young generation (under 29 years). Analysis of the subprograms suggests that the employment training program is strongly associated with the reduction in suicide rates.

Little is known about the association of personalized economic support with suicide prevention. One comparable program with “Supporting the Self-Reliance of Persons in Need” is the “Hope” project in the UK, which aims to support individuals facing financial, employment, or housing issues who were sent to emergency departments following self-harm or acute distress. Different from the self-reliance support in Japan, the “Hope” project also provides emotional support, including suicide and risk assessments, safety planning, as well as a listening, nonjudgmental, empowering, and compassionate approach. Meanwhile, the economic support is delivered from specialist advice workers as part of the supporting process (Farr et al., 2024). Compared with the “Hope” project, the “Supporting the Self-Reliance of Persons in Need” has the strength of wider application to the total population rather than high-risk groups, and practical subprograms providing economic support after advice services. However, emotional support is required for the self-reliance support program to improve mental health. Experimental studies on the “Hope” project found reduced suicide ideation and depression data in follow-up questionnaires (Barnes et al., 2018; Jackson et al., 2022), while further studies on larger samples are required to present the general population. There are also studies in other countries analyzing specific types of economic support programs. Studies from European countries focused on ALMP support that programs aim to improve the ability to get reemployed is accompanied by the effects on reducing individual depressive symptoms (Vuori & Vinokur, 2005) and local suicide rates (Mattei et al., 2019; Reeves et al., 2015; Stuckler et al., 2009). The studies on programs resolving food insecurity in the USA suggest that states with higher participation in the SNAP program are associated with lower total and male suicide rates (Rambotti, 2020). Compared to the literature, the current study benefits in 2 aspects. First, the “Supporting the Self-Reliance of Persons in Need” is open to the general population, which provides the opportunity to investigate the program at the population level. Second, the comprehensive subprograms of “Supporting the Self-Reliance of Persons in Need” provided an example of welfare program combination and the opportunity to compare different program types.

The results of the program are heterogeneous across age and gender. The largest association is observed among middle-aged individuals, especially males, aligning with literature that links unemployment and economic hardships with higher suicide risks in this demographic group (Li et al., 2011), as well as literature endorsing that economic interventions to prevent suicide are the most effective for this group (Mattei et al., 2019; Reeves et al., 2015; Shiroyama et al., 2021; Stuckler et al., 2009). Another reason might be attributed to the "employment ice age" generation entering middle age during the study period. The "employment ice age" generation in Japan refers to the generation that entered the labor market within the prolonged recession of the mid-1990s through the early 2000s, who reached the age of 25–45 during the study period of 2009–2015. The high unemployment rate at entry has a persistent negative effect lasting for over 10 years on the employment status and wage loss (Genda et al., 2010). The “Supporting the Self-Reliance of Persons in Need” targeting economic problems compensates the employment and income disadvantages of the "employment ice age" generation, which can partly explain the strong results on the middle-aged males. The association also extends to the young generation (under 29 years), even though financial reasons are less commonly cited for suicides in this group (MHLW, 2024d). In particular, the association exists for adolescents (under 20 years) (Table A.3), most of whom are students not yet in the workforce. The results on adolescents may reflect improved household financial security.

This study also finds that “employment training” is correlated with reduced suicide rates. The results suggest that efforts to promote employment might be more associated with reduced despair than direct financial benefits or advice at the population level. The employment training program is classified as an active labor market program (ALMP) designed to improve employability or increase earning capacity (Stuckler et al., 2009). Previous studies focus on the effects of increasing ALMP expenditure on reducing suicide rates (Mattei et al., 2019; Reeves et al., 2015; Stuckler et al., 2009). This study emphasizes the importance of understanding the types of interventions that are correlated with suicide prevention. Among the four employment support programs evaluated, only the employment training program is significantly correlated with lower suicide rates.

Several factors might tentatively explain the observed correlations among the subprograms. We need to understand that the interpretations should be regarded as hypotheses rather than causal pathways. Importantly, the estimates reflect population-level associations, not individual interventions. We should avoid the ecological fallacy of interpreting the effect on individual suicide risk from ecological studies. First, the employment training program has a relatively low number of applications compared with other subprograms, indicating a potential correlation between cities offering more comprehensive welfare services and the implementation of this program. In 2021, there were only 304 applications for the employment training program in Japan, in contrast to 4463 applications for the second-lowest and 10918 for the third-lowest subprogram. The availability of comprehensive subprograms may indicate better welfare provisions, which could be associated with lower suicide rates. Second, the variation in target groups among the four employment support programs may also contribute to differences in the outcomes observed. The target groups were separated based on their challenges in gaining employment. As shown in Table 2, the employment training program targets individuals who continue to face difficulties in finding mainstream jobs after receiving support from the employment preparation program, which in turn focuses on individuals experiencing severe challenges, such as socialization and communication. The other two subprograms are designed for individuals who can secure mainstream employment with some initial support. The employment training program provides multi-layered support following the employment preparation program. Third, the wages associated with the employment training program may also relate to its observed results. Unlike the employment preparation program, which does not involve formal job contracts, the employment training program introduces job contracts, sometimes accompanied by wages. Paid employment may provide a sense of self-reliance and material security, which are factors potentially associated with lower suicide rates. Further studies are required to understand the possible mechanisms by which employment training correlates with suicide reduction, as well as whether the intervention of employment training can be a personal intervention that reduces the suicide risk for individuals.

This study has several limitations, primarily related to data quality and availability. First, significant variability existed in monthly suicide deaths among cities, especially when separated by gender and age groups. During the study period, the proportion of months with a suicide rate of zero was 28.28 % for the total population. Yet, it increased to 60.35 % for the female population and 92.20 % for females under 29 years old. Although robustness tests using Poisson pseudo-maximum-likelihood estimators and estimations using quarterly or half-yearly suicide rates have been adjusted for the issue of zero-value outcomes, the results may still be biased owing to the city-month data structure.

Second, policy adoption limits quasi-experimental analyses. Pilot cities adopt the policy voluntarily rather than through random assignment. Pre-treatment differences in civic engagement, welfare provisions, or mental health infrastructure may lead to divergent suicide outcomes. What's more, the city-month data structure was further constrained by the short intervention period, which lasted only two years and three months. While using quarterly or half-yearly time intervals could reduce the variability in suicide rates, it would also decrease the sample size and precision of intervention timing, thereby limiting the precision of the estimates. Robustness checks conducted with quarterly or half-yearly data aggregations mitigate concerns about measurement instability.

Third, the binary indicator is difficult to reflect the heterogeneous design in pilot adoption before 2015. The analyses on the pilot cites are therefore focused on the presence of the program and the mandatory “self-reliance consultations” program. The subprogram application case data is only available after the national expansion in April 2015. Having complete records of application cases since the pilot program period would allow for a detailed analysis, adding variations in treatment based on dosage rather than binary categories. Additional data on program budget in the pilot period, if available, can also measure the heterogeneity in treatment intensity.

Finally, isolating the estimates of self-reliance support from other simultaneous suicide prevention measures remains challenging. Although the robustness test controlled for the Emergency Fund, the analysis is constrained by the absence of municipal-level data and the lack of observations beyond 2014, leaving open the possibility that the effects of other policies were not fully excluded. Future research that incorporates contemporaneous suicide-prevention related policies would help to strengthen the findings of this study.

## 5 Conclusion

This study suggests that the introduction of the personalized economic support program, “Supporting the Self-Reliance of Persons in Need”, in Japan might be one of the factors associated with the reduction in suicide rates during the 2010s. Its findings support the idea that, in addition to suicide prevention programs providing psychological assistance, welfare policies offering economic support through personalized consultations are associated with preventing suicides among the working-age population, particularly among males and the middle-aged. Furthermore, additional robustness checks, placebo tests, and heterogeneity analyses reinforce the core conclusion. A comparison of the content of different subprograms highlights those policies encouraging employment with multiple layers of support, rather than merely providing benefits to sustain living, may be more strongly linked to alleviating feelings of despair.

## Ethical statement

I declare that I follow all ethical guidelines for the paper

## Declaration of generative AI and AI-assisted technologies in the writing process

During the preparation of this study, the author used ChatGPT to revise the language. After using it, the author reviewed and edited the contents as needed and took full responsibility for the publication's content.

## Funding

This work was supported by JST 10.13039/501100025019SPRING, Grant Number JPMJSP2138.

## Declaration of competing interest

The authors declare the following financial interests/personal relationships which may be considered as potential competing interests: Xuanzi Zuo reports financial support, article publishing charges, equipment, drugs, or supplies, and travel were provided by 10.13039/501100002241Japan Science and Technology Agency. If there are other authors, they declare that they have no known competing financial interests or personal relationships that could have appeared to influence the work reported in this paper.

## Data Availability

Data will be made available on request.

## Footnotes

1 Note that during the study period, some cities underwent mergers, which means cities changed administrative borders. To construct a balanced panel data, the suicide rates are calculated to form the panel for 815 cities based on the 2020 territorial boundaries (Kondo, 2023). For instance, municipalities A and B merged to form municipality C in 2012. Using the 815 cities in 2020 territorial boundaries, only data for C are included in the study, while data for A and B are excluded. Before 2012, suicide rates for C are derived from aggregated suicide counts and populations of A and B. After 2012, C's suicide rates are calculated according to its own data.
