# Supplementary material for: Welfare policy and suicide: The role of “supporting the self-reliance of persons in need” program in Japan
Source: SSM Popul Health. 2025 Aug 19;31:101852. doi: 10.1016/j.ssmph.2025.101852 (PMC12398870; doi:10.1016/j.ssmph.2025.101852)
Supplement: Multimedia component 1 [file mmc1.docx]

**Glossary**

**Active labor market policy (ALMP):** A government approach to improving employment opportunities through training programs, job subsidies, and reintegration strategies.

**Difference-in-differences (DiD):** A statistical method used to estimate the effect of a policy intervention by comparing changes over time between a treated group and a control group.

**Economic hardship:** A situation where individuals face financial difficulties, leading to challenges in meeting basic living needs.

**Employment training program:** A subprogram under the self-reliance support initiative that provides skills training to enhance employability and reduce economic stress.

**Household finance support:** A welfare initiative providing financial management assistance to individuals struggling with economic stability.

**Parallel trends assumption:** A key requirement in DiD analysis stating that, in the absence of intervention, treated and untreated groups should exhibit similar trends over time.

**Public assistance act:** A Japanese legal framework that provides financial aid and social welfare services to individuals in need.

**Social isolation:** A condition where individuals experience a lack of social connections, increasing vulnerability to mental health issues and suicide.

**Suicide prevention policy:** Government strategies and programs aimed at reducing suicide rates through financial, mental health, and employment support.

**Supporting the self-reliance of persons in need:** A Japanese welfare initiative providing personalized consultations and financial assistance to individuals in economic distress.
