# Supplementary material for: Welfare policy and suicide: The role of “supporting the self-reliance of persons in need” program in Japan
Source: SSM Popul Health. 2025 Aug 19;31:101852. doi: 10.1016/j.ssmph.2025.101852 (PMC12398870; doi:10.1016/j.ssmph.2025.101852)
Supplement: Multimedia component 2 [file mmc2.docx]

**Appendices**

**
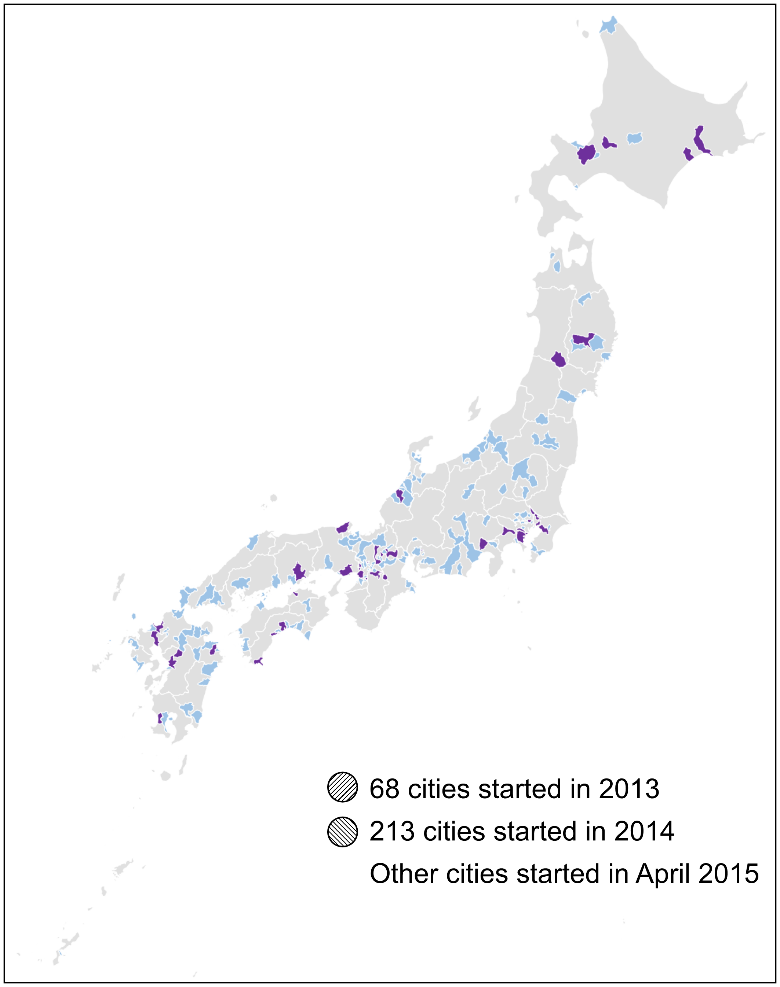
**

- 47 cities started in 2013
- 213 cities started in 2014

Other cities started in April 2015

**Figure A1**

*Process of Expanding the “Self-reliance Support for the Needy” Program in Pilot Cities*

******

**Figure A2**

*Event study with extended pretreatment periods*

Notes: The suicide data are drawn from the *Vital Statistics*, which provide gender-stratified municipality-year suicide counts before 2009. The horizontal axis in Figure A.2 indicates years relative to the introduction of self-reliance support in each city, ranging from -12 to +2 (i.e., 2003 to 2015). The vertical axis shows the estimated coefficients of each period on suicide rates. Consistent with the event study results in the main analysis, the extended pretreatment period up to 2003 shows that the estimated coefficients for male suicide rates remain close to zero (joint F-test *p* = 0.310). In contrast, the pretreatment estimates for females deviate from zero in some periods (joint F-test *p* = 0.015). These findings support the parallel trends assumption for males over the extended pretreatment window, while the deviations observed among females suggest caution in interpreting the female-specific results.

**Table A1**

*Poisson-pseudo-maximum-likelihood Estimates on Monthly Suicide Rates After Introducing Self-reliance Support in Pilot Cities, Stratified by Gender and Age*

| Age | All | All | ≤29 | ≤29 | 30−59 | 30−59 | ≥60 | ≥60 |
| --- | --- | --- | --- | --- | --- | --- | --- | --- |
| Gender | M | F | M | F | M | F | M | F |
| Support | -0.036** | -0.040* | -0.043 | -0.131* | -0.054** | -0.063* | -0.008 | 0.008 |
|  | (0.017) | (0.021) | (0.049) | (0.067) | (0.022) | (0.034) | (0.024) | (0.029) |
| Covariates | √ | √ | √ | √ | √ | √ | √ | √ |
| Fixed effects | √ | √ | √ | √ | √ | √ | √ | √ |

Notes: Robust standard errors are shown within parentheses. *** p<0.01, ** p<0.05, * p<0.1. A difference-in-differences analysis used the Poisson-pseudo-maximum-likelihood estimator (Correia et al., 2020). The estimation includes time-varying city characteristics and fixed effects for city, year, and month, estimated separately by gender and age groups. Each model yielded 68460 observations from 815 cities, 7 years, and monthly. Standard errors are clustered at the city level.

**Table A2**

*Two-stage Difference-in-Differences Estimator of Heterogeneity in Treatment Effects on Monthly Suicide Rates, After Introducing Self-reliance Support in Pilot Cities, Stratified by Gender and Age*

| Age | All | All | ≤29 | ≤29 | 30−59 | 30−59 | ≥60 | ≥60 |
| --- | --- | --- | --- | --- | --- | --- | --- | --- |
| Gender | M | F | M | F | M | F | M | F |
| Support | -0.387*** | -0.114 | -0.217** | -0.212** | -0.636*** | -0.056 | -0.235 | -0.100 |
|  | (0.113) | (0.070) | (0.110) | (0.084) | (0.186) | (0.106) | (0.235) | (0.131) |
| N | 68,460 | 68,460 | 67,872 | 60,900 | 68,460 | 68,292 | 68,460 | 68,460 |
| Covariates | √ | √ | √ | √ | √ | √ | √ | √ |
| Fixed effects | √ | √ | √ | √ | √ | √ | √ | √ |

Notes: Robust standard errors are indicated within parentheses. *** p<0.01, ** p<0.05, * p<0.1. A two-stage difference-in-differences analysis considers heterogeneity in treatment effects (Gardner, 2022). The estimation includes time-varying city characteristics and fixed effects for city, year, and month, which are estimated separately by gender and age groups. Standard errors are clustered at the city level.

**Table A3**

*Effects on Monthly Suicide Rates After Introducing Self-reliance Support in Pilot Cities for Youth Under 20 Years*

| Age | ≤19 | ≤19 | ≤19 |
| --- | --- | --- | --- |
| Gender | All | M | F |
| Support | -0.040** | -0.037 | -0.044** |
|  | (0.016) | (0.026) | (0.018) |
| Covariates | √ | √ | √ |
| Fixed effects | √ | √ | √ |

Notes: Robust standard errors are indicated within parentheses. *** p<0.01, ** p<0.05, * p<0.1. The difference-in-differences analysis included time-varying city characteristics and fixed effects for city, year, and month, which were estimated separately by gender and age groups. Each model yielded 68460 observations from 815 cities, 7 years, and monthly. Standard errors are clustered at the city level.
